# Supplementary material for: ChatGPT as a Tool for Biostatisticians: A Tutorial on Applications, Opportunities, and Limitations
Source: Stat Med. 2025 Oct 23;44(23-24):e70263. doi: 10.1002/sim.70263 (PMC12548020; doi:10.1002/sim.70263)
Supplement: Supplementary file 1 — Data S1. Supporting Information. [file SIM-44-0-s001.zip › Supplementary Materials Additional.zip!/3.5_Causal_Inferece/3.5_Causal_Chat10.pdf]

# - Combining statistical and causal thinking_10 - Clear memory request

Please clear memory.

I can't clear memory myself, but you can do it by clicking on your name or the three dots in the top-left corner, selecting **"Settings"**, then going to **"Personalization"** and clicking **"Clear ChatGPT memory."**

Let me know if you'd like to continue without referring to anything from previous chats—I can do that for this conversation.

Take the role of an expert biostatistician for this Chat

Understood. For this chat, I'll act as an expert biostatistician—ready to support you with statistical methodology, study design, data analysis, causal inference, regulatory standards, and interpretation of results. Please go ahead with your question or task.

I have data from a randomised clinical trial. I want to compare the efficacy of the experimental treatment with that of the control on overall survival in patients with non-small cell lung cancer. In this two-arm RCT, a considerable number of patients in the control arm switched to the experimental treatment after disease progression, while those in the experimental arm never switched.
My goal is to estimate the causal effect of sustained intake of the experimental treatment versus sustained intake of control on Overall survival, had the control group patients not been allowed to switch. Based on the information provided above, please define my target Estimand, following the guidance from the Estimand framework (ICH-E9 addendum)

Based on the scenario you described, here is a formal definition of your **target estimand** following the **ICH E9(R1) addendum** on estimands in clinical trials:

Estimand Definition:

Treatment:

- *Experimental intervention:* Assignment to the experimental treatment arm (with sustained intake).
- *Control intervention:* Assignment to the control treatment arm with sustained intake of control (i.e., no switching to experimental treatment allowed post-progression).

Population:

- Patients with non-small cell lung cancer eligible for and randomised in the clinical trial (i.e., the intention-to-treat population).

Variable (Endpoint):

- Overall survival (OS), defined as the time from randomisation to death from any cause.

Intercurrent Event(s) and Handling Strategy:

- **Treatment switching in the control arm (from control to experimental treatment after progression)** is the key intercurrent event.
- **Handling strategy:** *Hypothetical strategy* — we aim to estimate the OS that would have been observed **if patients randomised to control had not switched** to the experimental treatment.

Summary Measure:

- **Hazard ratio** (HR) for OS comparing sustained experimental treatment vs. sustained control (no switching), estimated using appropriate causal inference methods (e.g., inverse probability of censoring weighting [IPCW], rank-preserving structural failure time models [RPSFTM], or g-estimation).

Interpretation:

This estimand corresponds to a **hypothetical comparison** between two sustained treatment strategies: (1) remaining on experimental treatment throughout follow-up, and (2) remaining on control treatment throughout follow-up, assuming the switch in the control arm had not occurred. It isolates the effect of the **initial treatment assignment** as if the post-randomisation treatment switching had not been permitted.

Let me know if you'd like help translating this into a protocol or SAP table format, or choosing the most suitable method for estimation (e.g. IPCW vs. RPSFTM) based on your data.

> The switching behaviour is affected by a set of different baseline and time-varying covariates. Here, I want to assume that the time-varying confounders that influence the switching behaviour and Overall survival are also themselves affected by prior treatment intake.
> For e.g. ECOG score might not only affect whether patients should switch treatment or not, but its value might also be affected by prior treatment intake reflected by the switching behaviour.
> In this case, the analytical problem becomes more complex. Can you explain?

Yes, you're absolutely right—and you're describing a **classic case of time-varying confounding with treatment-confounder feedback**, which introduces **non-trivial complications** for causal inference.

Let me walk through **why** the analytical problem becomes more complex in your scenario.

---

1. Time-varying confounders that affect both treatment switching and survival

You correctly note that certain variables—like **ECOG performance status**, **disease progression**, or **tumour size**—are:

- **Predictors of switching**: e.g., a patient with worsening ECOG might be more likely to switch to the experimental treatment after progression.
- **Predictors of the outcome (OS)**: Poor ECOG is also associated with worse survival.
- **Updated over time**: They vary during follow-up.

---

2. Confounding affected by prior treatment (treatment-confounder feedback)

Some of these time-varying confounders are **themselves influenced by prior treatment history**. For instance:

- Experimental treatment might **delay functional decline** (affecting ECOG),
- This **improved ECOG** might then **reduce the likelihood of switching** (or delay it).

This feedback loop creates **treatment-confounder feedback**, meaning:

- Treatment affects a confounder,
- Which in turn affects future treatment,
- Which then affects the outcome.

This breaks the assumptions of **standard regression methods** or **time-dependent Cox models**, which would **adjust for time-varying covariates as if they were external (exogenous)**. Doing so can introduce **collider bias** or **block part of the treatment effect**, leading to **biased estimates**.

---

3. Analytical implications

This setup demands **specialised causal methods** that can handle:

- Time-varying treatments (like switching),
- Time-varying confounders affected by prior treatment.

These include:

Method	Key Idea	Strength
**Marginal Structural Models (MSMs) using Inverse Probability of Censoring Weighting (IPCW)**	Reweights individuals to create a pseudo-population where treatment is independent of time-varying confounders	Can handle time-varying confounding and treatment-confounder feedback
**G-estimation of Structural Nested Models (SNMs)**	Models counterfactual outcomes under different treatment regimes	Less reliant on correct modeling of treatment assignment
**G-formula (parametric or semi-parametric)**	Simulates outcomes under different hypothetical treatment regimes	Very flexible but requires full specification of the data-generating process

---

4. Why it matters for your estimand

Since your **target estimand** relies on a **hypothetical strategy** where no switching occurs in the control arm, you must account for the **fact that actual switching is both confounded and endogenous**. Without adjusting for time-varying confounders properly, your estimation of the causal effect of sustained treatment will be **biased**.

Summary

> The complexity arises because **time-varying covariates are both confounders and mediators**, making standard methods inappropriate. Appropriate methods (like MSMs with IPCW) are required to obtain unbiased causal estimates of the effect of sustained treatment vs. sustained control in the presence of treatment-confounder feedback.

Let me know if you'd like help specifying an MSM for your trial, defining the DAG, or estimating the weights.

Depict our research problem in a DAG

Here's a Directed Acyclic Graph (DAG) that represents your research problem involving:

- **Baseline and time-varying covariates**,
- **Treatment switching affected by these covariates**,
- **Time-varying confounders affected by past treatment** (i.e., treatment-confounder feedback),
- **Outcome: Overall survival (OS)**.

Textual DAG Representation

Let’s define the variables:

- `A0`: Randomised treatment assignment (0 = control, 1 = experimental)
- `L0`: Baseline covariates (e.g. age, ECOG at baseline)
- `L1(t)`: Time-varying covariates (e.g. ECOG, progression, tumour burden at time *t*)
- `A1(t)`: Actual treatment received at time *t* (0 = control, 1 = experimental) – switching can occur
- `Y`: Outcome (overall survival)

Now, the key **arrows (→)** in your DAG:

```java
L0       A0
 |       |
 |       v
 v       L1(t) <-----+
  \       |          |
   \      v          |
    --> A1(t) ------>|
          |          |
```

```
            v         v
                Y (Overall Survival)
```

Explanation of Arrows

- `` `L0 → A1(t)` ``: Baseline covariates influence treatment switching.
- `` `L0 → Y` ``: Baseline covariates influence overall survival.
- `` `A0 → L1(t)` ``: Initial treatment influences future health status (e.g. ECOG).
- `` `A0 → A1(t)` ``: Randomised treatment affects likelihood and timing of switching.
- `` `L1(t) → A1(t)` ``: Time-varying health status affects whether patients switch.
- `` `L1(t) → Y` ``: Time-varying covariates affect survival.
- `` `A1(t) → L1(t+1)` ``: Current treatment influences future health status (treatment-confounder feedback).
- `` `A1(t) → Y` ``: Actual treatment received affects survival.

Key Feature: Treatment–Confounder Feedback

- `` `A1(t) → L1(t+1) → A1(t+1)` `` forms a feedback loop where past treatment affects time-varying covariates, which in turn affect future treatment decisions.
- This violates the assumptions of standard regression methods and necessitates **G-methods** like **MSMs**, **g-formula**, or **SNMs**.

Would you like me to draw this DAG visually or annotate it for inclusion in a protocol or paper?

I would like to apply the inverse probability of censoring weighting (IPCW) approach to address the bias introduced by switching following the methodology discussed in the following paper: Robins JM, Finkelstein DM. Correcting for noncompliance and dependent censoring in an AIDS clinical trial with inverse probability of censoring weighted (IPCW) log-rank tests. Biometrics. 2000;56(3):779–88.
Briefly summarise the key points from this paper

Certainly. The seminal paper by **Robins & Finkelstein (2000)** lays the theoretical and methodological foundation for using **Inverse Probability of Censoring Weighting (IPCW)** to estimate causal treatment effects in the presence of **noncompliance** and **dependent censoring**, particularly when treatment switching occurs. Here are the **key points**:

---

1. Motivation: Treatment switching as a source of bias

- In randomised trials, patients may **switch treatments post-randomisation**, violating the intent-to-treat (ITT) assumption.

- If switching is **informative**—i.e., influenced by prognostic time-varying covariates—naive per-protocol or as-treated analyses can be **biased**.
- Standard methods like time-dependent Cox models are **inappropriate** when covariates affecting switching are also affected by prior treatment (treatment-confounder feedback).

---

2. Conceptual solution: Estimating effects under a hypothetical strategy

- The goal is to estimate the effect of **sustained treatment strategies**, e.g. "What would survival have looked like if no patients switched?"
- This requires correcting for **dependent censoring** introduced by artificially **censoring patients at the time of switch** and reweighting the remaining patients to create a pseudo-population that is representative.

---

3. IPCW: The core method

- IPCW adjusts for dependent censoring using **stabilised weights** based on the inverse of the estimated probability of remaining uncensored (i.e. not switching).
- These probabilities are estimated from a **model for the censoring process**, typically using pooled logistic regression or Cox models with baseline and time-varying covariates.

Weight formula:

Let $C(t)$ be an indicator of remaining uncensored at time t, and $\bar{L}(t)$ the history of covariates:

$$\text{Weight}_i(t) = \prod_{s=0}^{t} \frac{P(C_i(s) = 1 \mid \bar{L}_i(s), \bar{A}_i(s))}{P(C_i(s) = 1 \mid \bar{L}_i(s), \bar{A}_i(s), \text{past survival})}$$

- Weights are then used in **weighted survival analyses** (e.g., weighted Kaplan–Meier or Cox/log-rank tests).

---

4. Application: AIDS Clinical Trial

- The authors applied IPCW to data from an AIDS trial with treatment switching and found **substantial bias** in conventional analyses.
- The IPCW-adjusted log-rank test better estimated the **causal effect of initial treatment assignment**, had patients adhered to their assigned treatment.

---

5. Assumptions

IPCW relies on several key assumptions:

1. **No unmeasured confounding**: All variables predicting switching and outcome are measured.
2. **Correct model specification**: The model for censoring must be correctly specified.
3. **Positivity**: There is a non-zero probability of remaining uncensored for all covariate patterns.

6. Limitations and practical considerations

- IPCW can yield **unstable weights** when probabilities of remaining uncensored are very small.
- It's crucial to **truncate extreme weights** and **diagnose model fit and overlap**.
- IPCW estimates a **hypothetical estimand** and is **sensitive to assumptions and model choice**.

Summary

Robins & Finkelstein (2000) introduced IPCW as a principled way to recover **causal effects under hypothetical sustained treatment strategies** in the presence of **treatment switching and time-varying confounding**. The method is robust to treatment-confounder feedback, provided its assumptions are met.

Let me know if you'd like help applying IPCW to your data or constructing the censoring model.

Could you elaborate more on the research question Robins and Finkelstein were trying to answer in their paper, mentioning the comparator arms, possible intercurrent events, outcome definition and population under study.

Certainly. In their 2000 paper, **Robins and Finkelstein** addressed a causal inference problem arising in the context of an **AIDS clinical trial**, where **treatment switching** occurred post-randomisation. Here's a more detailed breakdown of the **research question** and study context using the **Estimand framework language (ICH E9-R1)**:

---

1. Clinical Context

- The study reanalysed data from the **AIDS Clinical Trials Group Protocol 019 (ACTG 019)**, a **randomised controlled trial (RCT)**.
- Participants: HIV-positive individuals with **CD4+ T-cell counts >500/mm³**.
- Treatment arms:
  - **Zidovudine (AZT)**: active antiretroviral therapy.
  - **Placebo**

---

2. Treatment and Comparator

- **Treatment (A1)**: Assignment to AZT at baseline.
- **Comparator (A0)**: Assignment to placebo.
- However, in practice, **patients in the placebo group were allowed to switch to AZT** after certain clinical criteria were met (e.g., decline in CD4+ counts or AIDS-defining illness).

---

3. Intercurrent Events

- The **key intercurrent event** is **treatment switching**: patients initially assigned to placebo **crossed over to AZT** during follow-up based on evolving health status.
- Switching was **non-random** and depended on **time-varying prognostic factors** such as CD4 count, which is both:
  - A **predictor of switching** and of the **outcome**,
  - **Influenced by prior treatment**, leading to **treatment-confounder feedback**.

---

4. Outcome Definition

- The primary outcome was **time to AIDS or death**, a typical survival endpoint.
- Observed from **randomisation until the first occurrence** of AIDS or death from any cause.

---

5. Population

- The **intent-to-treat (ITT) population**: all participants randomised to either AZT or placebo.
- The goal was to estimate the **causal effect** of sustained AZT use compared to sustained placebo **had switching not occurred**—that is, under a **hypothetical strategy**.

---

6. Research Question (Causal Estimand)

> What would the difference in survival time (free of AIDS or death) have been between AZT and placebo, **had all patients remained on their originally assigned treatment**, and **no switching** occurred?

This translates to a **hypothetical estimand**:

- **Treatment:** Sustained AZT vs. sustained placebo.
- **Population:** HIV+ adults with CD4 > 500 cells/mm^3 enrolled in the ACTG 019 trial.
- **Variable:** Time to AIDS or death.
- **Intercurrent Event Strategy: Hypothetical strategy** — assume no treatment switching occurs.
- **Summary Measure:** Differences in survival curves or log-rank tests (e.g., weighted Kaplan–Meier), using **IPCW** to correct for **dependent censoring due to switching**.

Conclusion

Robins and Finkelstein sought to answer a clinically meaningful and policy-relevant question: *What is the effect of AZT versus placebo, assuming patients adhered to their assigned treatment for the duration of follow-up?*
This required developing IPCW methods to adjust for the **informative censoring** introduced when patients were censored at the time of treatment switching, and for the **complex confounding structure** arising from time-varying covariates affected by prior treatment.

Would you like help translating this into your own trial context or aligning your IPCW implementation with the structure used in this paper?

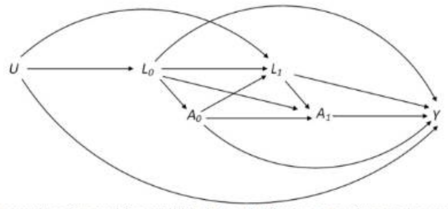

Fig. 1 Graphical representations of our research focus and the bias of naive methods for time-varying confounding in the presence of treatment-confounder feedback. This Directed acyclic graph (DAG) represents the post-progression period for patients in the control arm. Following disease progression, the decision as to whether or not a patient in the control group continues to take its treatment (A_0) or (A_1) depends on predictive factors measured at or before the time of disease progression (L_0). Again, we assume that treatment intake (A_0) affects outcome (Y) and influences a set of TVCs at a later time-point (L_1). These covariates are also assumed to impact the clinician's decision as to whether or not a patient should subsequently change treatments (A_1) introducing time-varying confounding and treatment-confounder feedback through L_1

Attached, I provide you with a directed acyclic graph (DAG) that represents our research problem.
To simplify this DAG and take into consideration this deterministic information provided below, I restrict my DAG to the post-progression period for patients in the control arm only and consider two follow-up time points, 1 & 2.
Here A(k) is deterministically 0 if A(k-1)=0 AND L(k-1) (reflecting progression status)=0 (no progression).
Following disease progression, the decision as to whether or not a patient in the control group continues to take their treatment (A0 or A1) depends on predictive factors measured at or before the time of disease progression (L0). These covariates are also assumed to impact the clinician’s decision as to whether or not a patient
should subsequently change treatments (A1), introducing time-varying confounding and treatment confounder feedback through L1.
I will emphasise again that in our case scenario, patients in the control group do not switch unless they progress, and all patients in the experimental group never switch.
I still want to estimate in my outcome model the causal effects of sustained treatment interventions (always taking control versus always taking experimental) under a hypothetical strategy where control patients do not switch treatment.
I want you to use this DAG as a basis for performing the appropriate IPCW analyses and provide me with the analysis R-script.
You may assume that the dataset is in long format, with variables: id, time (in months and increases with increments of 1), death indicator, switching indicator, baseline covariates
repeated over time points, time-varying covariates at each time pt.
Again, we assume that treatment intake (A0) affects outcome (Y) and influences a set of TVCS at a later time-point (L1).

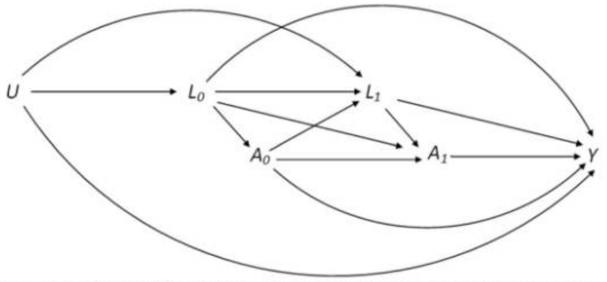

Fig. 1 Graphical representations of our research focus and the bias of naive methods for time-varying confounding in the presence of treatment-confounder feedback: This Directed acyclic graph (DAG) represents the post-progression period for patients in the control arm. Following disease progression, the decision as to whether or not a patient in the control group continues to take its treatment (A_0) or (A_1) depends on predictive factors measured at or before the time of disease progression (L_0). Again, we assume that treatment intake (A_0) affects outcome (Y) and influences a set of TVCs at a later time-point (L_1). These covariates are also assumed to impact the clinician's decision as to whether or not a patient should subsequently change treatments (A_1) introducing time-varying confounding and treatment-confounder feedback through L_1

Thank you for confirming the structure. Based on the DAG, your goal is to estimate the **causal effect of sustained control vs. experimental treatment on overall survival** under a **hypothetical strategy** where **no switching** occurs in the control arm. You want to use **IPCW** to adjust for the bias introduced by **informative switching**, which is influenced by both baseline and time-varying covariates that are themselves affected by prior treatment.

Below is a tailored **R script** for IPCW estimation of survival under your specified assumptions, using your **long-format dataset**.

🔧 R Code for IPCW Estimation

```r
r


# Required libraries
library(survival)
library(dplyr)
library(ipw)

# Assumes your data is named `trial_data` with:
# id, time (months), death (1/0), switch (1/0), A (treatment actually received),
# baseline covariates (e.g., age, ECOG0, etc.), time-varying covariates (e.g., ECOG_t, prog_t)

# Step 1: Create artificial censoring at switch
trial_data <- trial_data %>%
  mutate(censored = ifelse(switch == 1, 1, 0))

# Step 2: Estimate probability of remaining uncensored (not switching)
# Fit pooled logistic regression model to estimate weights
weight_model <- glm(
  censored ~ age + ECOG0 + prog_t + ECOG_t + time,  # adjust as needed
  data = trial_data,
  family = binomial()
)

# Predict probabilities
trial_data$prob_uncensored <- 1 - predict(weight_model, type = "response")

# Step 3: Calculate stabilized weights
# Numerator model: only includes baseline covariates
numerator_model <- glm(
  censored ~ age + ECOG0,
  data = trial_data,
  family = binomial()
)

trial_data$prob_uncensored_numerator <- 1 - predict(numerator_model, type = "response")

# Stabilized weights
trial_data$sw <- with(trial_data, prob_uncensored_numerator / prob_uncensored)

# Step 4: Artificially censor at switch time
```

```
trial_data_censored <- trial_data %>%
  group_by(id) %>%
  mutate(cum_switch = cumsum(switch)) %>%
  filter(cum_switch == 0 | time == min(time[cum_switch == 1])) %>%
  ungroup()

# Step 5: Use weighted survival model (e.g., Cox)
# Use the actual treatment assigned at baseline as exposure
cox_ipcw <- coxph(Surv(time, death) ~ A, data = trial_data_censored, weights = sw)

summary(cox_ipcw)
```

✅ Key Assumptions and Notes

- This approach adjusts for **informative censoring at the time of switching**.
- The **pooled logistic model** accounts for time-varying covariates that influence switching.
- You can **truncate extreme weights** to stabilise the estimation, e.g. `trial_data$sw <- pmin(trial_data$sw, 10)`.
- This yields an **IPCW-adjusted hazard ratio** comparing the hypothetical strategy of no switching versus sustained experimental treatment.

Let me know if you'd like:

- Help defining the censoring model formula more precisely based on your actual covariates,
- Code for checking positivity or inspecting weight distributions,
- An implementation using `survey::svykm()` or `ipw::ipwpoint()` functions,
- IPCW under discrete time intervals using survival curves instead of Cox.
